# A comparative study of *Mentha longifolia* var. *asiatica* and *Zygophyllum arabicum* ZnO nanoparticles against breast cancer targeting Rab22A gene

**DOI:** 10.1371/journal.pone.0308982

**Published:** 2024-08-30

**Authors:** Iqra Bashir, Erum Dilshad

**Affiliations:** Department of Bioinformatics and Biosciences, Faculty of Health and Life Sciences, Capital University of Science and Technology (CUST), Islamabad, Pakistan; Institute for Biological Research, University of Belgrade, SERBIA

## Abstract

Breast cancer is the most frequently diagnosed cancer worldwide, and the incidence rate has increased enormously over the last three decades. Rab proteins are members of the Rab GTPase superfamily. The aberrant function of these proteins leads to the development of tumors. *Mentha longifolia var*. *asiatica* and *Zygophyllum arabicum* have been known for their therapeutic potential for ages. The present study aimed to synthesize ZnO nanoparticles encapsulated with the extracts of *M*. *longifolia* var. *asiatica* and *Z*. *arabicum* and evaluating their therapeutic potential against breast cancer, targeting the Rab22A gene and its protein. UV-Vis spectrophotometer showed characteristic absorbance peaks at 295 nm and 345 nm for *Z*. *arabicum* and *M*. *longifolia* var. *asiatica* ZnONPs, respectively. The FTIR bands of *Z*. *arabicum* nanoparticles suggested the presence of aldehydes, alcohols, and polyols whereas bands of *M*. *longifolia* var. *asiatica* ZnONPs suggested the presence of carboxyl groups, hydroxyl groups, alkynes, and amines. SEM revealed the size of *Z*. *arabicum* ZnO NPs to be 25 ± 4 nm with a spherical shape as compared to nanoparticles of *M*. *longifolia* var. *asiatica* having a size of 35 ± 6 nm with a hexagonal shape. EDX determined the elemental composition of both particles. The cytotoxicity of both plant extracts and respective NPs was determined against the MCF-7 breast cancer cell line, which was found to be significant with an IC_50_ value of 51.68 μM for *Z*. *arabicum* and 88.02 μM for *M*. *longifolia* var. *asiatica* ZnO compared to plant extracts (64.01 μM and 107.9 μM for *Z*. *arabicum* and *M*. *longifolia* var. *asiatica)*. The gene expression and protein levels of Rab22A were decreased in nanoparticle-treated cells as compared to the control group. The apoptotic role of synthesized nanoparticles against the MCF-7 cell line was also determined by the expression of apoptotic pathway genes and proteins (bax, caspase 3, caspase 8 and caspase 9). All samples showed significant apoptotic activity by activating intrinsic and extrinsic pathway genes. The activity of *Z*. *arabicum* was more eminent as compared to *M*. *longifolia* var. *asiatica* which was evident by the greater expression of studied genes and proteins as determined by Real-time qPCR and ELISA. This is the first-ever report describing the comparative analysis of the efficacy of *Z*. *arabicum* and *M*. *longifolia* var. *asiatica* ZnONPs against breast cancer.

## Introduction

Cancer is classified into a group of diverse diseases distinguished by dysregulation in multiple gene expressions that control normal cellular proliferation and differentiation, leading to an imbalance in apoptosis and replication [1]. The incidence of cancer is growing manyfold worldwide. It is thought to be the major cause of death and the utmost barrier against increasing the expectancy of life in the 21^st^ century. According to WHO statistics, there were approximately 9.6 million deaths attributed to cancer annually [2]. As of 2021, breast cancer in women has surpassed lung cancer, making it the leading cause of cancer incidence globally. It accounts for over 2.3 million cases annually. This accounts for 24.5% of all cancer cases and about 685,000 deaths, which is a 30% rise from WHO estimates in 2012 [3].

The Ras superfamily is comprised of over 150 different small GTPases that are members of distinct families. The Ras family (36 members), Rho family (20 members), and Rab family (more than 60 members) are the subfamilies of small GTPases in this superfamily which show the best characterization [4]. These proteins take part in vital cellular signalling networks that control gene expression, cytoskeletal structure, protein and vesicle transport inside cells, and cell proliferation [5]. The Ras superfamily member Rab22A gene is found at 20q13.32 chromosomal position. According to the literature, Rab22A participates in the endocytic pathway at several levels. Additionally, endocytic recycling and uptake control the makeup of the plasma membrane receptors. In literature, it is reported that Rab22A also mediates the trans-golgi network and early endosome trafficking [6].

The use of nanotechnology in the fight against cancer generated a new domain called "Cancer Nanotechnology," which is defined as the application of nanotechnology in the detection, diagnosis, imaging, and treatment of cancer. Metallic nanoparticles (MNPs) have a lot of potential for medicinal applications in cancer treatment [7]. Among these materials, zinc oxide (ZnO) nanoparticles are considered a good candidate for biocompatible applications due to their biocompatibility, economic advantage, and low toxicity [8]. In fact, ZnONPs show toxicity against cancer cells via reactive oxygen species generation and destruction of mitochondrial membrane potential, which leads to the activation of caspase cascades followed by apoptosis of cancerous cells. Moreover, ZnONPs have also been used as an effective carrier for targeted and sustained delivery of various plant bioactive and chemotherapeutic anticancerous drugs into tumor cells [9].

Despite all the advancements which have been made in the treatment of cancer, the concern regarding the side effects of these therapies is still there which also damages the healthy cells of the body, making these therapies a poor choice [10]. Moreover, these conventional therapies also face the problem of developing resistance after some doses. Furthermore, other limitations are also there owing to their nonspecific targeting, inability to invade tumors, low solubility and damage to the immune system and other healthy tissues of the body, therefore, offering a low survival rate. The technology of nanomedicine has opened new horizons for novel cancer treatments through the encapsulation of therapeutic compounds or drugs in nanoparticulate materials and their targeted delivery by passive permeation and active internalization mechanisms into tumors [9].

The synthesis of nanoparticles from biological organisms (plants, bacteria, algae and fungi) proved to be very cost-effective and environmentally friendly. Phytochemicals have a range of biological effects, which include antioxidant, anti-microbial, anti-inflammatory and anticancer. Preclinical research has demonstrated the enormous potential of phytochemicals to treat or prevent breast cancer [11]. According to the literature, the utilization of plant’s secondary metabolites and the prevention of tumor formation by modification of several cell signalling pathways are directly related [12]. Numerous phytochemicals found in functional foods control apoptosis, proliferation, angiogenesis, inflammation, invasiveness and metastasis events linked to malignant transformation. Numerous studies have revealed that phytochemicals have a major role in controlling epigenetic alterations and metabolic reprogramming which are two crucial processes for the initiation, development, and advancement of cancer [12].

*Zygophyllaceae* is a diverse plant family with around 27 genera, of which the genus *Zygophyllum* has 80 species [13]. *Zygophyllum arabicum* (L.) Christenh. & Byng is one of the species of this genus. The plants of the genus *Zygophyllum* have been used to treat different ailments, including diabetes, hypertension, rheumatism, and microbial infections. Moreover, several reports confirm the diverse pharmacological activities of these plants. They are important for their anti-inflammatory activities and are used as Ayurvedic medicine in the Indian subcontinent. Different phytochemicals found in the plant, including flavonoids, saponins and essential oils help cure stomach problems, skin diseases and cancer [14, 15]. The genus *Mentha* is another important genus and holds a very important position in the family *Lamiaceae*. This genus typically comprises 25–30 species, and they are widely found in various regions of the world. They are found abundantly in the South Africa, Australia and temperate regions of Eurasia [16]. *Mentha longifolia* var. *asiatica* Borris. Rech.f. known as Asian mint is a member of the family ***Lamiaceae***. Various kinds of bioactive compounds and essential oils have been isolated from this species which show cytotoxic activity [17, 18]. In addition to this, *Mentha* spp. has long been used as a folk remedy for different ailments such as ulcers, nausea, flatulence, bronchitis, liver complaints and colitis due to presence of phytochemicals [18, 19]. The current study was aimed to synthesize ZnONPs of both *Z*. *arabicum*a and *M*. *longifolia* var. *asiatica* and compare their cytotoxic potential against breast cancer cell line MCF7 by studying their impact on the Rab22A gene along with apoptotic pathway genes and proteins.

## Materials and methods

All the experiments were conducted according to national and international guidelines for conducting plant research. The project was approved by the Institutional Ethical Review Committee with approval reference No. Ref: CUST-2022/1.

### Preparation of plant extract

*Z*. *arabicum* was collected from Gujjar Khan, Rawalpindi, and *M*. *longifolia* var. *asiatica* was collected from the surroundings of Quaid-i-Azam University Islamabad. Both plants were identified from the herbarium of Quaid-i-Azam University Islamabad, with a specimen voucher no. that was submitted to the herbarium. For the preparation of plant extracts 0.2 g of leaves of both plant species were thoroughly washed with distilled water to remove any debris and impurities. Aerial parts of plants were boiled in 100 mL of water for about 30 minutes. After boiling, both extracts were allowed to cool at room temperature. After cooling, extracts were filtered using Whatman filter paper and labeled. Extracts were saved in the refrigerator (4°C) for the synthesis of zinc oxide nanoparticles [20].

### Preparation and characterization of ZnO nanoparticles

A 5 mM solution of zinc acetate was prepared in distilled water. The prepared salt solution was reduced with plant extract in a ratio of 9:1. The solution was kept in the dark for 2 hours. Centrifugation of vials was carried out at maximum speed for 30 minutes. Pellets were rinsed 5–7 times and the whole process was repeated 3 times. Particles were dried in a hot air oven at 50–60°C. Prepared nanoparticles of *Z*. *arabicum* and *M*. *longifolia* var. *asiatica* were characterized by using UV-Vis spectroscopy for their optical properties [20, 21]. Samples were placed in the cuvettes and absorption was recorded in the range of 250–400 nm. The morphology of synthesized nanoparticles was determined by SEM (JEOL-JSM-6490LATM) at voltage of 20 Kv with a frequency of 2838 cps (max). The average size of synthesized nanoparticles along with S. E. was determined after finding the individual particle sizes in the field. Energy-dispersive X-ray spectroscopy that was coupled with an SEM was carried out for synthesized ZnONPs to determine the elemental composition and their proportion in the sample. FTIR was performed for the analysis of surface chemistry of synthesized ZnONPs. For that purpose, dried solutions at 75°C, were characterized at the range of 4000–400 cm^-1^ by using the KBr pellet method. Moreover, topological features were determined by X-ray diffraction. The energy of the beam in the 10–20 KeV range caused the X-rays emission from samples. The electron beam moved across the samples and images were obtained for the synthesized ZnONPs [21]. Furthermore, the crystallite parameters of prepared nanoparticles were calculated using following Debye Scherrer’s relation, D = 0.9λ/β × cosθ [22].

### Cell viability assay

The tumorigenic human breast cancer cell line (MCF-7) was obtained from ATCC with ATCC number CCL-2™ and managed according to the recommendations of ATCC. The cytotoxic effect of plant extracts and respective zinc oxide nanoparticles was assessed by MTT assay. Cells (1*10^4^) were seeded in 96 well plate for 24 hours containing 20μL of RPMI growth medium. Various concentrations (20, 40, 60, 80 and 100 μM) of ZnONPs and plant extracts were prepared and the cells were treated with these concentrations over different incubation periods (24 h and 48 h) according to the reported protocol. The treated and control cells were incubated for 4 hours at 37°C after adding 150 μL of fresh medium and 50 μL of MTT solutions. The absorbance was recorded at 570 nm [23].

### Gene expression studies by real-time qPCR

The expression of targeted gene Rab22A, bax and apoptotic pathway genes (caspase 3, caspase 8 and caspase 9) was studied by real-time qPCR according to reported methodology [24, 25]. The experiment was run with IC_50_ (concentration in μM) of both plant extracts and their respective ZnONPs. After washing all treated and untreated cells with phosphate buffer saline (pH 7.2), 1 mL of RNX-plus solution (SinaClone, Iran) was applied to each cell in accordance with the kit’s instructions. Following the confirmation of the cDNA synthesis by the standard agarose gel electrophoresis, the cDNAs were kept at -20°C in preparation for Real-Time qPCR investigations. The target gene expression was standardized using β actin as a reference gene and relative expression was frequently utilized. It was expressed as a fold change from the β actin level and quantified using the ΔCt technique.

### Determination of protein levels by ELISA

The protein levels of the enzyme’s caspases, bax and Rab22A were measured using ELISA kits, following the manufacturer’s instructions (Thermo-Fisher Scientific) as triplicates. The experiment was run with IC_50_ (concentration in μM) of both plant extracts and their respective ZnONPs. The ELISA kit’s instructions were followed for preparing the cell lysates. A horseradish peroxidase conjugated secondary antibody was used to detect the proteins from the cell lysate that bound selectively to the primary antibody. Protein concentrations were then assessed at 450 nm [25].

### Statistical analysis

Each experiment was conducted in triplicate and the standard deviation and standard error were calculated and a T-test was performed. A value of p ≤ 0.05 was deemed statistically significant, and quantitative data were presented as mean ± SE. GraphPad Prism V.5 was utilized for performing two-way ANOVA.

## Results and discussion

### Preparation and characterization of ZnO nanoparticles

**Fig 1:**
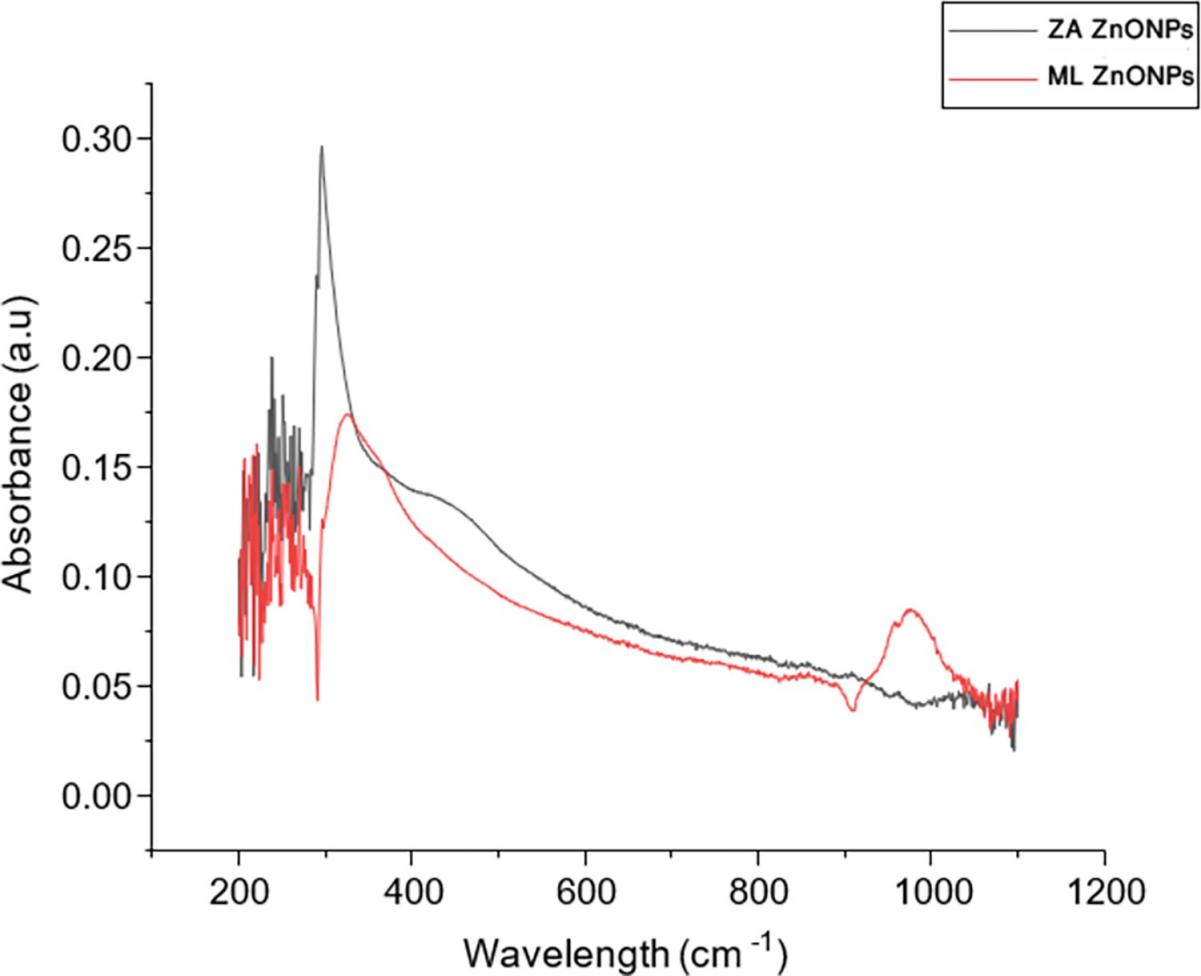
UV-Vis spectroscopy. The spectra show the absorption peaks of ZnONps of *Z*. *arabicum* and *M*. *longifolia* var. *asiatica*.

The change of colour from brown to light yellow and brown to transparent white indicated the synthesis of ZnONPs of *Z*. *arabicum* and *M*. *longifolia* var. *asiatica* respectively. This was further validated by UV-Vis spectroscopy. The characteristic peak for ZnONPs synthesized from *Z*. *arabicum* was observed at 295 nm (Fig 1). This is in accordance with the characteristic peak shown by ZnONPs prepared from *Zygophyllum Coccineum* and *Fagonia cretica* extracts (250–400 nm) [26, 27]. The characteristic absorbance peak of ZnONPs synthesized from *M*. *longifolia* var. *asiatica* was observed at 345 nm (Fig 1). In a previous report, ZnO NPs prepared from *Nigella sativa* also showed an absorbance peak at 370 nm under UV-Vis spectroscopy [22]. These results are also supported by previously synthesized ZnO nanoparticles of *Mentha longifolia* where an absorbance peak was observed at 365 nm [8]. Moreover, no additional peak was observed at 500 nm confirming that synthesized ZnO nanoparticles are hydroxyl free.

**Fig 2:**
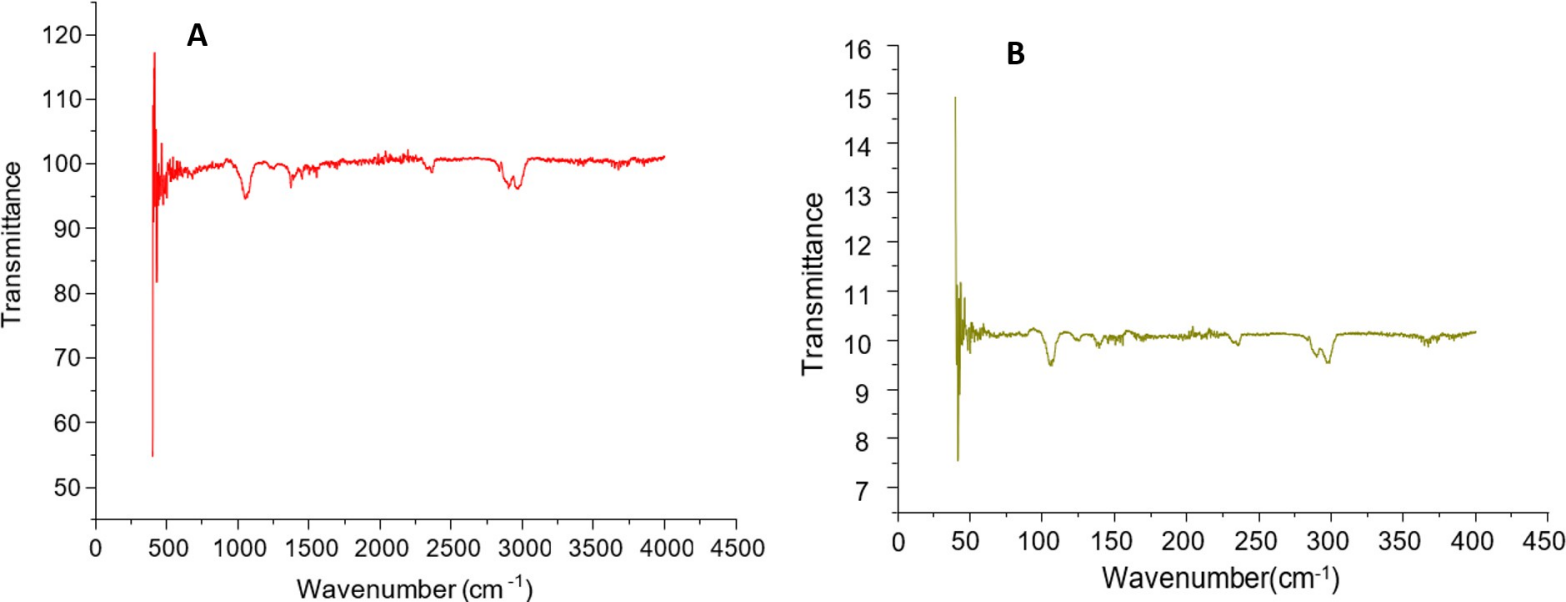
FTIR analysis. (A) FTIR spectra of ZnO NPs synthesized from *Z*. *arabicum*, (B) FTIR spectra of ZnO NPs synthesized from *M*. *longifolia* var. *asiatica*.

FTIR spectrophotometer was used to perform FTIR analysis in order to identify the different biomolecules that were present in the aqueous extracts of both plants and were involved in the creation of nanoparticles. The FTIR spectroscopy was done to validate the functional groups present in both plants which were responsible for the reduction of zinc ions into ZnONPs. The major bands of the FTIR spectrum of *Z*. *arabicum* were observed at 2970, 2888, 2832, 2370, 2318, 1379 ad 1067 cm^-1^. The minor bands were observed at 2160, 1561, 1375, 873, 600 and 594 cm^-1^ (Fig 2A). The major bands corresponding to 2970–2832 cm^-1^ indicated the presence of C-H group of aldehydes suggesting presence of saturated compounds. The bands at 2370–2318 cm^-1^ indicated the presence of hydroxyl O-H group demonstrating the presence of alcohols. A distinct band at 1067 cm^-1^ indicated the presence of the C-O group of polyols. These groups are responsible for the reduction of metallic zinc. These results are in accordance with the previous reports where major bands were recorded at 2200–3000 cm^-1^ in the synthesis of ZnONPs from *Zygophyllum Coccineum* [27]. In another report, comparable results were observed [26]. FTIR spectroscopy was also carried out for ZnONPs synthesized from *M*. *longifolia* var. *asiatica*. The major bands were observed at 3649, 2989, 2969, 2900, 2365, 2025 and 1383 cm^-1^. The minor bands were observed at 1558, 1570, 1370, 1261 and 852 cm^-1^ (Fig 2B). The major bands indicated the presence of O-H, C-H and C-O functional groups. The major band at 3649 cm^-1^ showed the presence of bending of the N-H group indicating the presence of proteins. The bands at 2989, 2969 and 2900 cm^-1^ indicated the presence of C-O groups whereas bands at 2365 and 2025 cm^-1^ indicated stretching bond C-C of alkynes. The band at 1383 cm^-1^ corresponds to the iso-propyl functional group of hydroxyl flavones. In a previous study, green synthesis of ZnO nanoparticles from *M*. *longifolia* also showed FTIR spectra in the range of 400–4000 cm^-1^ with similar results [28].

**Fig 3:**
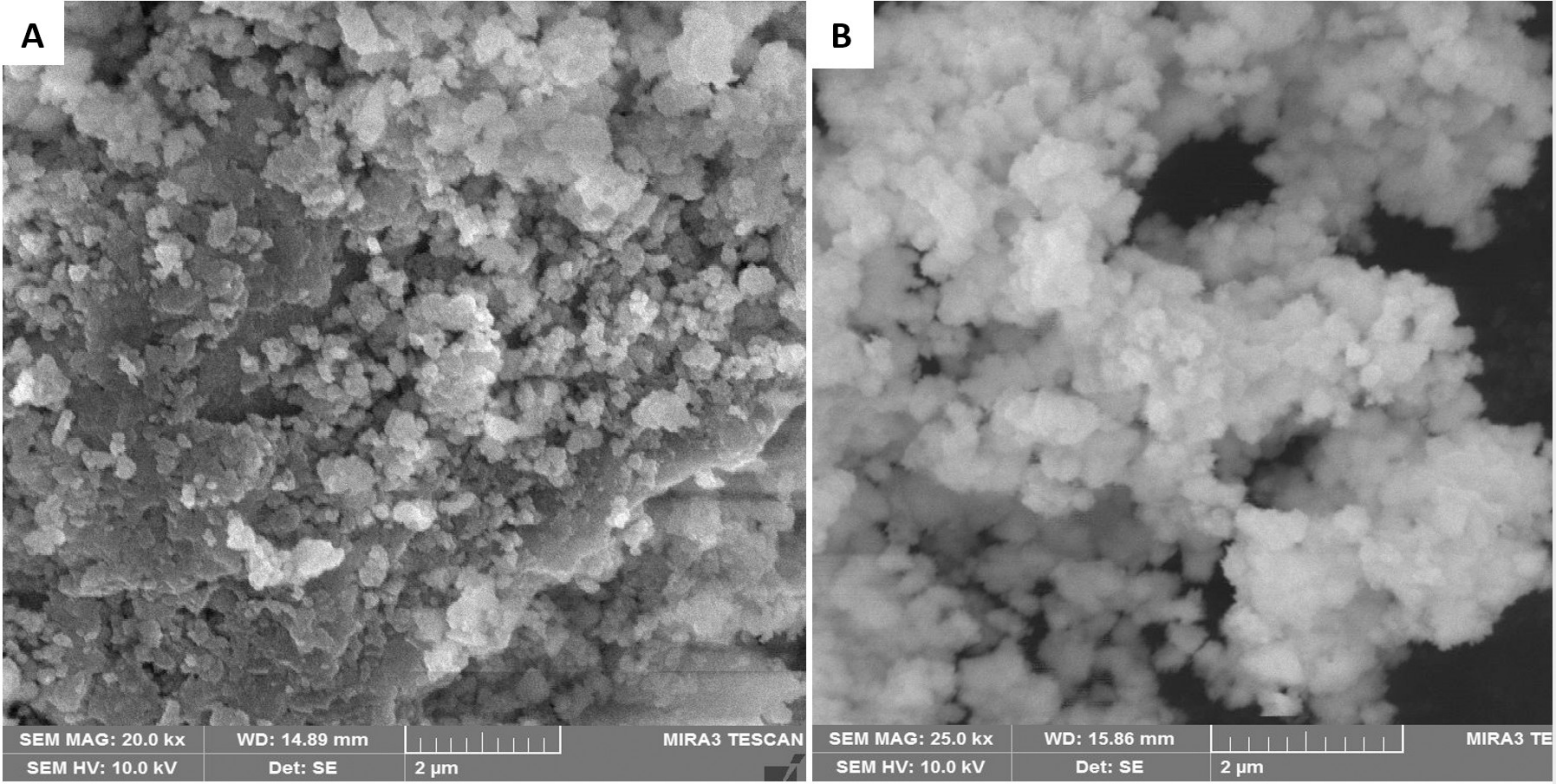
SEM analysis. (A) SEM images of *Z*. *arabicum* ZnO nanoparticles, (B) SEM images of *M*. *longifolia* var. *asiatica* ZnO nanoparticles.

Scanning electron microscopy (SEM) was used to determine the size and shape of synthesized nanoparticles of *Z*. *arabicum* and *M*. *longifolia* var. *asiatica* (Fig 3A & 3B). The micrographs showed that ZnONPs were in the nanoscale range and they had uniform size distribution. The size of nanoparticles synthesized from *Z*. *arabicum* was 25 ± 4 nm (Fig 3A) and they were spherical in shape as compared to nanoparticles of *M*. *longifolia* var. *asiatica* which had a size range of 35 ± 6 nm (Fig 3B) and they were hexagonal in shape. These results correlate with the previous report where ZnONPs of *Z*. *coccineum* leaves showed a size of 35.569 nm with a spherical shape [29]. Moreover, previously synthesized Ag and Au nanoparticles of *Mentha longifolia* were found to have the size of 10.23 ± 2 and 13.45 ± 2 nm, respectively, with a spherical shape [30].

**Fig 4:**
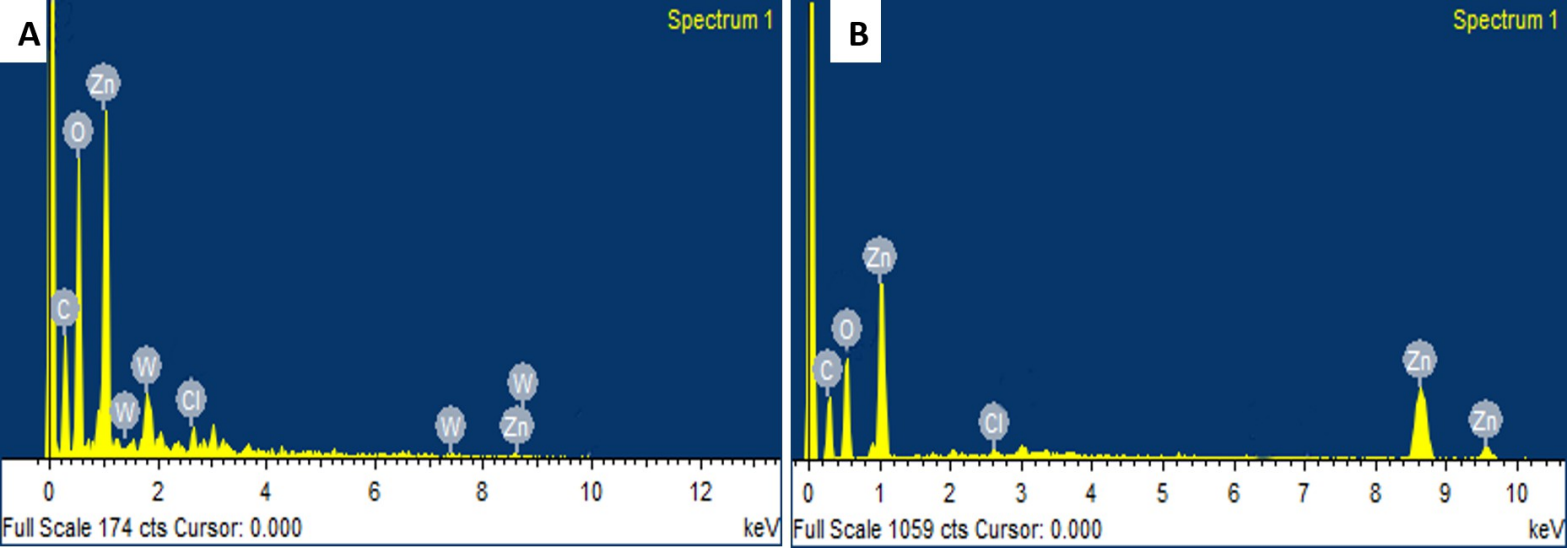
EDX analysis. (A) EDX spectra of *Z*. *arabicum* ZnO nanoparticles, (B) EDX spectra of *M*. *longifolia* var. *asiatica* ZnO nanoparticles.

**Fig 5:**
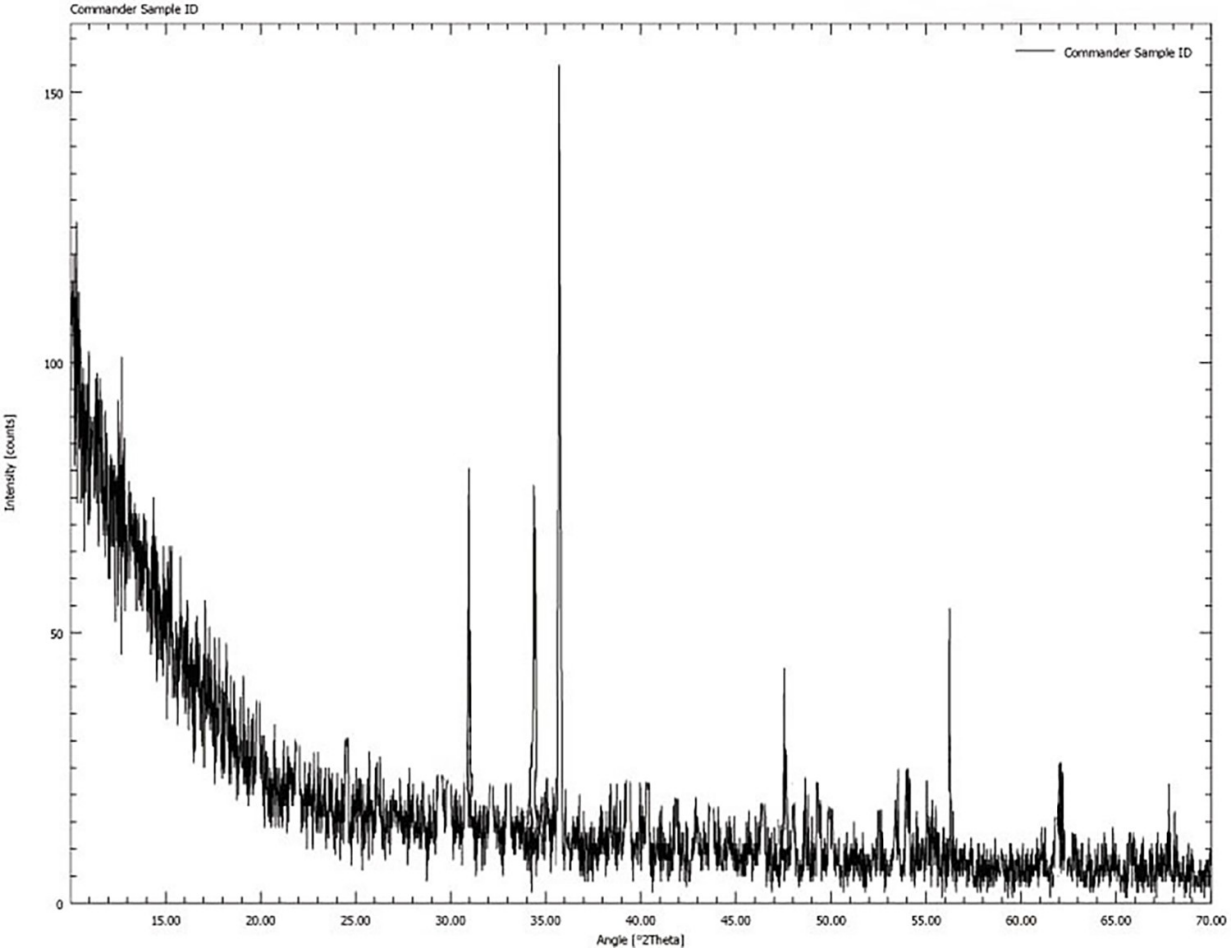
XRD analysis. The XRD pattern of synthesized ZnO nanoparticles. The intensity on the vertical axis is measured in counts per second (CPS), and the diffraction angle (2 theta) measured is taken along the horizontal axis. The value of wavelength in angstrom (a.u.) is also indicated.

Using energy-dispersive X-ray spectroscopy coupled with a scanning electron microscope, the chemical and elemental composition of the synthesized ZnONPs of both plants was ascertained [26]. The EDX spectrum suggested that the Zn is the primary component along with oxygen in both samples without the presence of any contaminants as shown by the primary peaks in Fig 4A & 4B. Similar findings were presented by the ZnONPs of *Mentha longifolia* and *Z*. *coccineum* [8, 27]. Moreover, XRD was performed for the ZnONPs of both plants to determine the crystalline alignment phase composition and phase identification. The XRD pattern of ZnONPs was observed by using index POWDER-X software and matched with standard JCPDS, 36–1451 data. The result showed the diffraction peaks of ZnONPs at 31.34°, 34.50°, 36.32°, 47.60°, 56.68°, 62.94° (Fig 5) matching with lattice parameters of (100), (002), (101), (012), (110), (013), which indicated the crystalline nature of the nanoparticles [8, 22, 31].

### Cell viability assay

**Fig 6:**
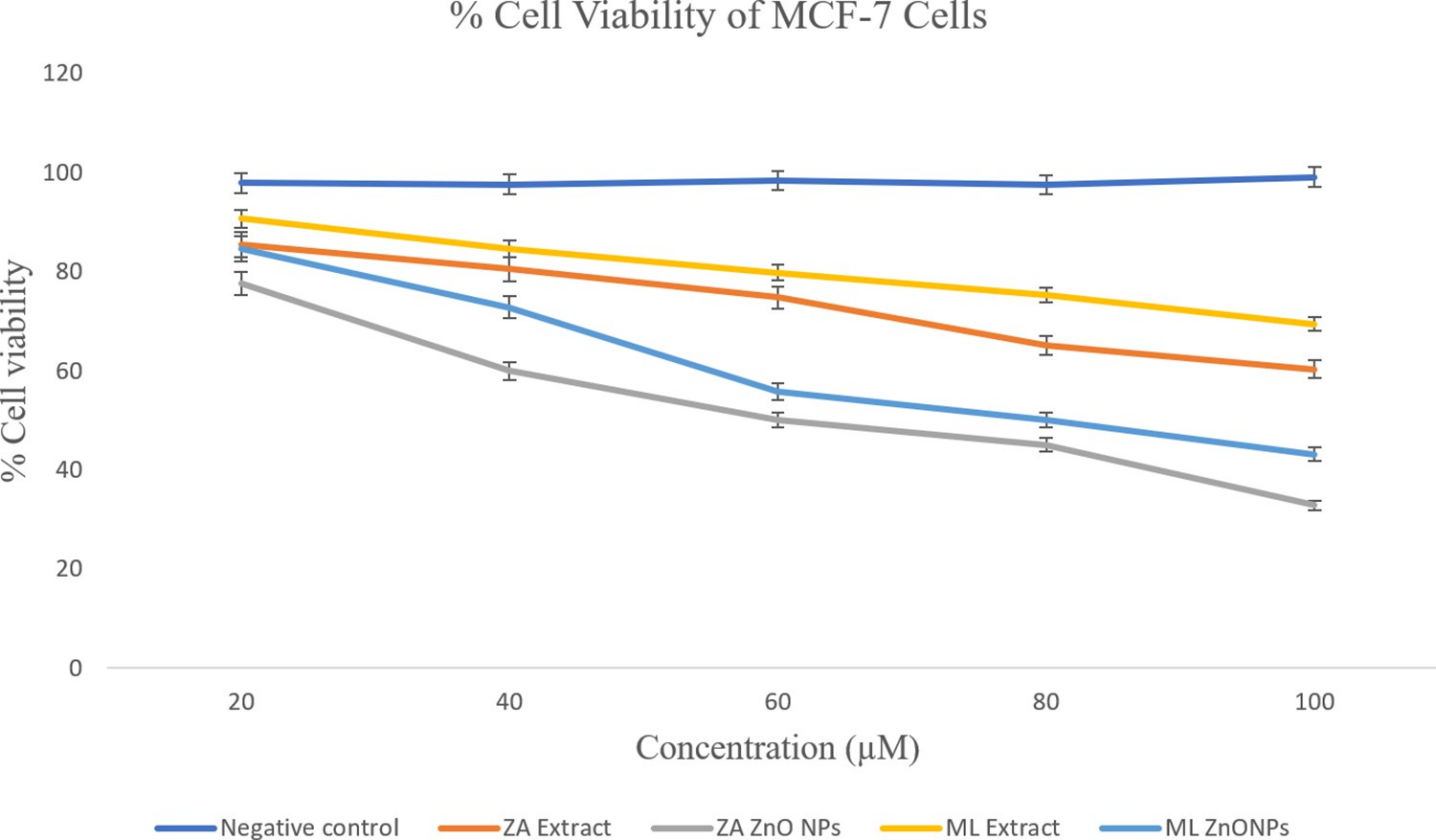
Cell viability (MTT) assay results. Cell viability of MCF-7 cell line treated with plant extract and ZnO NPS of *Z*. *arabicum* and *M*. *longifolia* var. *asiatica*. Error bars indicate standard error (SE) of three means, asterisk represents the significant difference in data compared with control at *P < 0.05, **P < 0.01 and ***P< 0.001. ZA = *Zygophyllum arabicum*, ML = *Mentha longifolia* var. *asiatica*.

The cytotoxic potential of ZnONPs synthesized from *Z*. *arabicum* and *M*. *longifolia* var. *asiatica* was assessed against the MCF-7 breast cancer cell line. Different concentrations (20, 40, 60, 80 and 100 μM) of nanoparticles and plant extract showed potent cytotoxicity. The percentage of cell viability of the MCF-7 cell line exposed to extracts and ZnONPs of *Z*. *arabicum* and *M*. *longifolia* var. *asiatica* showed variations. Incubation of MCF-7 cells with 20–100 μM of ZnONPs and respective plant extracts showed decreased cell viability in a dose dose-dependent manner. With increasing concentrations of plant extracts and NPs, cell viability was found to be decreased (Fig 6). The lowest cell viability of treated cells was observed at 100 μM of ZnONPs of *Z*. *arabicum*, which was 32.8%, whereas for the extract of *Z*. *arabicum* it was 60.3%. On the other hand, in the case of treatment with the extract and ZnONPs of *Mentha longifolia* var. *asiatica*, the cell viability was found to be 69.4% and 43.2% respectively. ZnONPs of studied plant species were found to be more effective in lowering the viability of breast cancer cell line as compared to the respective plant extracts. The IC_50_ value of *Z*. *arabicum* and *M*. *longifolia* var. *asiatica* ZnONPs was also found to be decreased as compared to that of their respective extracts. Moreover, *Z*. *arabicum* extract and nanoparticles showed higher cytotoxicity (IC_50_ 64.01 μM and 51.68 μM respectively) as compared to those of *M*. *longifolia* var. *asiatica* extract and respective nanoparticles (IC_50_ 107.9 μM and 88.02 μM respectively).

The cytotoxicity results were also found statistically significant (Table 1), which shows the analysis of variance for the factors affecting the viability of MCF-7 cells. All of these factors, including type of sample (extracts and their ZnONP), concentration of these samples and interaction of both of these factors were found to have a significant impact on the viability of MCF-7 cells with p-value <0.0001.

**Table 1:** Analysis of variance for factors affecting the viability of MCF-7 cells.

Source of Variation	Df	Sum-of-squares	Mean square	F-Value	P Value	Significant
**Interaction**	16	2605	162.8	42.28	<0.0001	Yes
**Types of sample**	4	18200	4550	1182	<0.0001	Yes
**Concentration**	4	6417	1604	416.7	<0.0001	yes
**Residual**	50	192.5	3.850			

In another report, the extract of *Z*. *coccineum* showed significant cytotoxic activity against breast cancer (MCF-7), colorectal (HCT-116) and liver cancer (HepG2) cell lines as reported in literature [32]. Moreover, green synthesis of Ag NPS from *Fagonia cretica* showed significant cytotoxicity with IC_50_ values 0.101 ± 0.004, 0.177 ± 0.03 and 0.434 ± 0.022 mg/mL against MCF-7, HepG2 and HUH-7 cell lines respectively [33]. In a particular study, synthesis of silver and gold NPs prepared from *M*. *longifolia* also exhibited significant cytotoxic activity [30]. Moreover, in a previous report, silver nanoparticles of *Mentha asiatica* were also found effective against the breast cancer cell line (MCF-7) with an IC_50_ of 11.8 μM [23].

### Gene expression and protein analysis

**Fig 7:**
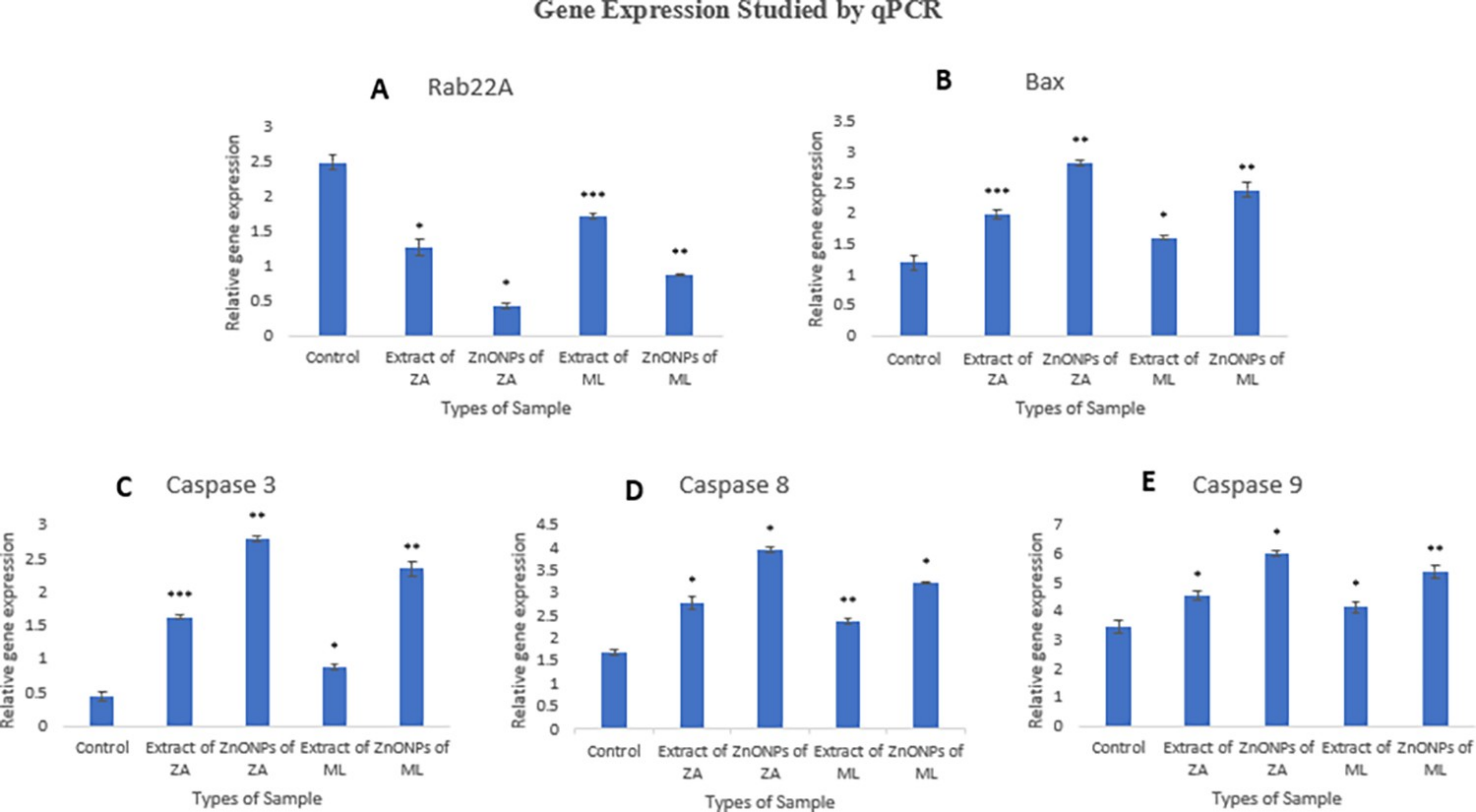
Gene expression studies. Level of Rab22A, bax and caspase 3, caspase 8 and caspase 9 genes determined by real time q-PCR. Error bars indicate standard error (SE) of three means, asterisk represents the significant difference in data compared with control at *P < 0.05, **P < 0.01 and ***P< 0.001. ZA = *Zygophyllum arabicum*, ML = *Mentha longifolia* var. *asiatica*.

The apoptotic role of plant extracts and synthesized ZnONPs was also studied by the expression of Rab22A, bax and caspases genes as illustrated in Fig 7. *Z*. *arabicum* nanoparticles and respective plant extract showed greater potential in down-regulating the Rab22A gene expression as compared to the nanoparticles and plant extract of *M*. *longifolia* var. *asiatica*. Rab22A which is primarily involved in endocytic recycling and membrane trafficking of endosomes is a major drug target for treating multiple malignancies. The level of this gene significantly declined in the breast cancer cells treated with ZnONPs and plant extracts of both plant species as compared to untreated cells. This may be attributed to the fact that this gene is considered as an oncogene and participates in the carcinogenesis of breast cancer.

It is also reported that aberrant expression of Rab22A is also observed in hepatocellular carcinoma and melanoma [24]. These results correlate with the previous findings in which knockdown of Rab22A led to inhibiting lung cancer cell migration and invasion. Rab22A is also involved in clathrin-independent endocytosis (CIE) by recycling of CD147 protein. This protein maintains the integrity of the plasma membrane and intracellular signalling. Silencing of the Rab22A gene by short interfering RNAs inhibits the recycling of CDI47 protein. Effective recycling of this protein is a hallmark of lung and breast cancer [6].

The present study also observed the up-regulation of the bax gene, which is involved in the apoptotic pathway. Plant extract and ZnONPs of *Z*. *arabicum* showed greater apoptotic potential by up-regulating bax gene expression as compared to plant extract and NPs of *M*. *longifolia* var. *asiatica* in treated MCF-7 breast cancer cells in comparison with the control group. The greater bax/bcl-2 gene ratio decreases the resistance faced by apoptotic genes thereby inducing apoptosis. The caspase 3, caspase 8 and caspase 9 showed higher expression as well which decipher the role of extrinsic and intrinsic pathways of apoptosis in nanoparticles mediated toxicity [25]. Cells treated with plant extract and ZnONPs of *Z*. *arabicum* showed greater expression of caspase 3, caspase 8 and caspase 9 as compared to those treated with extract and NPs of *M*. *longifolia* var. *asiatica*. These results indicate that *Z*. *arabicum* has more cytotoxic potential by up-regulating the apoptotic genes in comparison to *M*. *longifolia* var. *asiatica*. These findings correlate with the previous reports where ZnO nanoparticles synthesized by the sol-gel technique showed greater caspase 8 activity after 24 hours of treatment in MCF-7 cells [34]. Previously, the apoptotic role of ZnO nanoparticles was also evaluated in HepG2, BEAS-2B and A549 cancer cells. Their findings suggest that mRNA and protein levels of the bax gene were increased as compared to the bcl-2 gene against all the cancer types studied by producing reactive oxygen species [35]. Apoptosis is a highly regulated and evolutionarily conserved programmed cell death that is essential for normal physiological processes like embryogenesis and adult tissue homeostasis. It is also widely recognized for its function as a mechanism that suppresses cancer growth [36].

**Fig 8:**
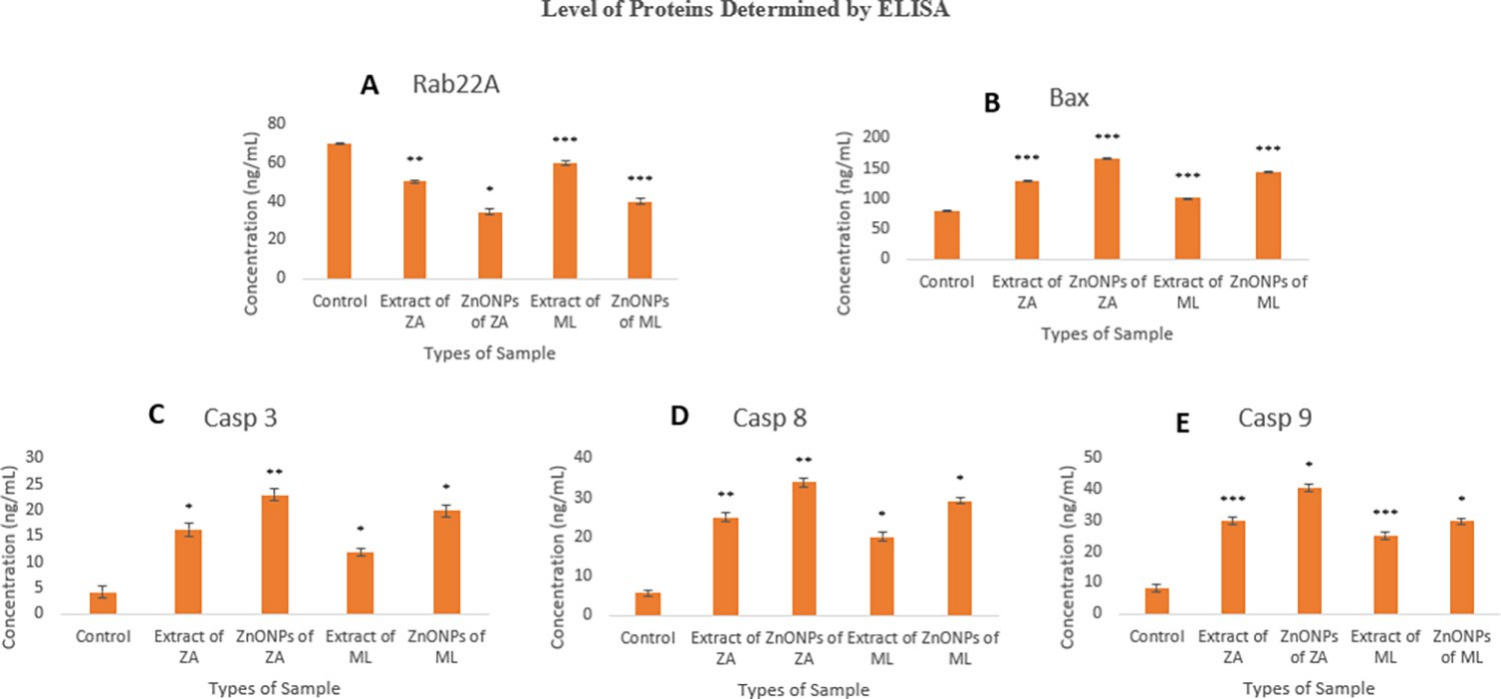
Determination of protein levels. Rab22A, bax and caspases protein levels determined by ELISA. Error bars indicate standard error (S.E.) of three means, asterisk represents the significant difference in data compared with control at *P < 0.05, **P < 0.01 and ***P< 0.001. ZA = *Zygophyllum arabicum*, ML = *Mentha longifolia* var. *asiatica*.

ELISA was used to determine the protein levels of the aforementioned genes. The level of Rab22A, bax, and initiator caspase (caspase 9) and executioner caspases (caspases 3 and caspase 8) proteins was studied in MCF-7 cells treated with *Z*. *arabicum* and *M*. *longifolia* var. *asiatica* ZnONPs and plant extracts (Fig 8). It was observed that Rab22A protein level was decreased while bax protein and caspases protein levels were increased in treated cells as compared to the control group. The results also showed that the level of studied proteins was higher in the cells treated with *Z*. *arabicum* as compared to *M*. *longifolia* var. *asiatica* in comparison to the control group. This confirmed the role of plant extract and ZnONPs in the activation of cell death in cancer cells by activating a series of caspase reactions. These results are supported by the previous reports in which ZnONPs up-regulated the testis tissue’s protein levels of bax, cleaved caspase-3, and cleaved caspase-8, and down-regulated bcl-2 protein levels, suggesting that ZnONPs may trigger apoptosis in the testis [37]. The increased expression of these caspases (caspase 3, caspase 8, and caspase 9) at the mRNA level and protein level showed that plant-mediated ZnONPs have induced programmed cell death at a significant level.

## Conclusion

The present study described the green synthesis of ZnONPs of *Z*. *arabicum* and *M*. *longifolia* var. *asiatica* which after characterization were tested against the breast cancer cell line (MCF-7). The highest cytotoxicity was observed at the highest tested concentration (100 μM) of these nanoparticles and their respective plant extracts. The Rab22A gene was studied as a drug target and was found to be down-regulated in the treated cells. The level of proteins of the Rab22A gene was also found to be decreased there. Moreover, the apoptotic role of synthesized nanoparticles was also evaluated by observing the enhanced gene expression and protein levels of the bax gene along with caspase enzymes. The anticancer activity of synthesized ZnONPs and plant extracts of *Z*. *arabicum* was found to be comparatively higher than that of *M*. *longifolia* var. *asiatica* extract and respective ZnONPs having more potential to be used in the development of anticancer drugs against breast cancer. However, further research is needed to determine the toxicity of these nanoparticles in animal models for the development of nanomedicine against breast cancer.

## Supporting information

S1 File Raw data files of cell viability assay, gene and protein analysis, statistical analysis and SEM images are uploaded as supporting information. (ZIP)
