# Supplementary figures and images for: A comparative study of Mentha longifolia var. asiatica and Zygophyllum arabicum ZnO nanoparticles against breast cancer targeting Rab22A gene
Source: PLoS One. 2024 Aug 30;19(8):e0308982. doi: 10.1371/journal.pone.0308982 (PMC11364221; doi:10.1371/journal.pone.0308982)

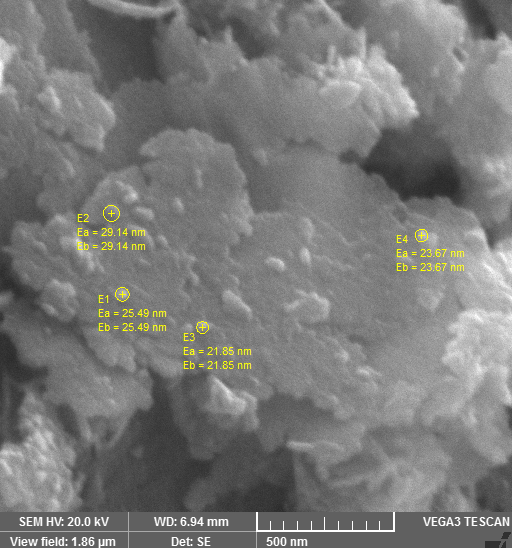

Supplement: S1 File — (ZIP) [file pone.0308982.s001.zip › SEM Image of M. L ZnONPs.tif]

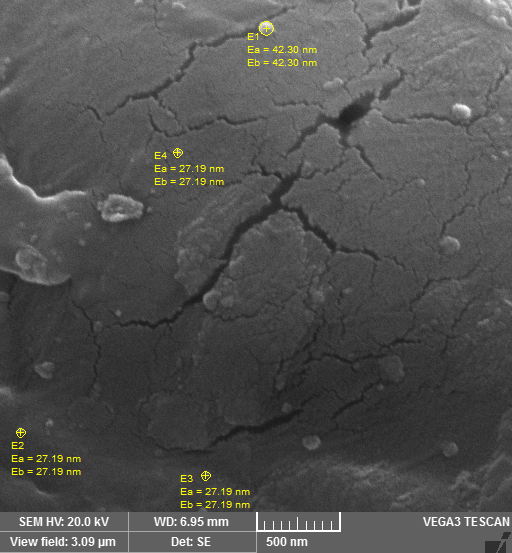

Supplement: S1 File — (ZIP) [file pone.0308982.s001.zip › SEM image of Z. A ZnONPs.tif]
